# Quantification of measurable residual disease in patients with multiple myeloma based on the IMWG response criteria

**DOI:** 10.1038/s41598-021-94191-8

**Published:** 2021-07-22

**Authors:** Kentaro Narita, Daisuke Miura, Takafumi Tsushima, Toshiki Terao, Ayumi Kuzume, Rikako Tabata, Masami Takeuchi, Kosei Matsue

**Affiliations:** Division of Hematology/Oncology, Department of Medicine, Kameda Medical Center, 929 Higashi-chou, Kamogawa-shi, Chiba, 296-8602 Japan

**Keywords:** Haematological cancer, Myeloma

## Abstract

Stringent complete response (sCR) is defined as a deeper response than complete response (CR) in multiple myeloma. Whether achieving sCR correlates with better survival remains controversial. We evaluated the outcomes in patients with intact immunoglobulin multiple myeloma (IIMM) and light chain multiple myeloma (LCMM) who achieved a very good partial response (VGPR) or better. Multicolour flow cytometry was used to assess the depth of response. LCMM patients with sCR had significantly lower measurable residual disease (MRD) levels than those with CR (median MRD: 7.9 × 10^–4^ vs. 5.6 × 10^–5^, *P* < 0.01). Nonetheless, no significant difference was observed in MRD levels across the responses in groups of patients with IIMM (VGPR vs. CR: 3.5 × 10^–4^ vs. 7.0 × 10^–5^, *P* = 0.07; CR vs. sCR: 7.0 × 10^–5^ vs. 5.4 × 10^–5^, *P* = 0.81. In accordance with MRD levels, the median overall survival of patients with sCR was significantly longer (sCR, CR, VGPR; not reached, 41 months, and 58 months, respectively; VGPR vs. CR, *P* = 0.83; CR vs. sCR, *P* = 0.04) in LCMM, but not in IIMM (sCR, CR, VGPR; not reached, 41 months, and not reached, respectively; VGPR vs. CR, *P* = 0.59; CR vs. sCR; *P* = 0.10). Our results show that sCR represents a deeper response that correlates with longer survival in patients with LCMM, but not IIMM.

## Introduction

Serum free light chain (FLC) measurements are widely used to diagnose and assess treatment response in patients with multiple myeloma (MM). Owing to recent developments in effective novel agents and their combinations for MM treatment, an increasing number of patients achieve complete response (CR), leading to their prolonged survival^1–3^. In the latest International Myeloma Working Group (IMWG) response criteria, CR with a normal FLC ratio (rFLC) was classified as stringent CR (sCR) and categorized as a deeper response than CR^4^. Several studies, including our previous report, describe the advantage of achieving sCR to prolong the time to next treatment (TNT) or overall survival (OS). However, in other studies, achievement of sCR had no impact on OS or TNT^5–10^. Owing to these contradictions, the impact of achieving sCR on the survival of patients with MM remains controversial. However, many studies show that measurable residual disease (MRD)-negativity after treatment could improve progression-free survival (PFS) or TNT^11–13^.

MRD-negativity evaluated using multicolor flow cytometry (MFC), allele-specific oligonucleotide-polymerase chain reaction (ASO-PCR), or next-generation sequencing (NGS) is broadly used in many clinical trials as an optimal biomarker of better survival in patients with MM^12,14–16^. MFC is the most widely used technique to evaluate MRD in clinical trials and routine clinical practice because it is cost-effective and quicker than ASO-PCR or NGS^13,17^. ‬ Despite sCR being a deeper response than CR, its influence in terms of MRD on longer survival in patients with symptomatic MM has not yet been demonstrated, especially in real-world clinical practice. Furthermore, the impact of rFLC normalization at the time of CR may differ between intact immunoglobulin multiple myeloma (IIMM) and light chain multiple myeloma (LCMM). Therefore, MRD levels in the same IMWG response categories may not be the same for IIMM and LCMM.‬ MRD assessment allows for significant variability in the depth of response among patients with MM, even in those who achieve CR or sCR. Here, we present the relationship between the depth of MRD levels and IMWG responses in patients with IIMM and LCMM who achieved very good partial response (VGPR) or better.‬‬‬‬‬‬‬‬‬‬‬‬‬‬‬‬‬‬‬‬‬‬‬‬‬‬‬‬‬‬‬‬‬‬‬

## Results

The baseline characteristics were similar across patients achieving VGPR, CR, and sCR from LCMM and IIMM (Tables 1 and 2). Similarly, there were no differences in the initial treatment regimens between patients with LCMM and IIMM achieving VGPR, CR, and sCR (Tables S1 and S2). The median observation period for all patients was 41.0 months (range 3–143). Median levels of serum FLC at diagnosis were 4490 mg/L and 375 mg/L in LCMM and IIMM, respectively (*P* < 0.01).

**Table 1:** Baseline characteristics of patients with LCMM.

LCMM (n = 42)	All patients	VGPR (n = 7)	CR (n = 7)	sCR (n = 28)	p-value
Age, median (range)	69.5 (46–86)	66 (54–86)	73 (50–86)	69 (46–86)	0.9
**Sex, n (%)**					1
Male	27 (64.2)	2 (28.5)	2 (28.5)	11 (39.2)	
Female	15 (35.7)	5 (71.4)	5 (71.4)	17 (60.7)	
ECOG PS: 0, 1, n (%)	26 (61.9)	5 (71.4)	6 (85.7)	15 (53.5)	0.13
**Diagnosis, n (%)**					0.26
Kappa light chain only	26 (61.9)	3 (42.8)	6 (85.7)	17 (60.7)	
Lambda light chain only	16 (38.1)	4 (57.1)	1 (14.2)	11 (39.2)	
**ISS, n (%)**					1
I	9 (21.4)	1 (14.2)	2 (28.5)	6 (21.4)	
II	8 (19.0)	1 (14.2)	1 (14.2)	6 (21.4)	
III	25 (59.5)	5 (71.4)	4 (57.1)	16 (57.1)	
**R-ISS, n (%)**					0.86
I	7 (16.6)	0	2 (28.5)	5 (17.8)	
II	19 (45.2)	4 (57.1)	3 (42.8)	12 (42.8)	
III	15 (35.7)	3 (42.8)	2 (28.5)	10 (35.7)	
Not available	1 (2.3)	0	0	1 (3.5)	
High risk cytogenetic abnormalities	5 (11.9)	1 (14.2)	1 (14.2)	3 (10.7)	1
β2-microglobulin (mg/L), median (range)	7.85 (1.0–56.7)	8.3 (3.0–28.4)	6.1 (1.1–56.7)	7.8 (1.0–34.6)	0.56
Albumin (g/dL), median (range)	3.85 (2.6–5.2)	4.0 (2.9–5.2)	3.4 (2.6–4.5)	3.9 (2.6–5.0)	0.33
LDH (U/L), median (range)	210 (136–810)	220	200	210	0.90
Creatinine (mg/dL), median (range)	1.48 (0.47–11.48)	3.48	1.20	1.61	0.60

LCMM: Light chain multiple myeloma; ECOG: Eastern Cooperative Oncology Group; PS: performance status; ISS: international staging system; R-ISS: revised international staging system.

**Table 2:** Baseline characteristics of patients with IIMM.

IIMM (n = 121)	All patients	VGPR (n = 27)	CR (n = 8)	sCR (n = 86)	p-value
Age, median (range)	71 (30–87)	75.0 (43–87)	75.0 (58–87)	68.5 (30–87)	0.07
**Sex, n (%)**					
Male	65 (53.7)	18 (66.6)	4 (50.0)	43 (50.0)	0.32
Female	56 (46.2)	9 (33.3)	4 (50.0)	43 (50.0)	
ECOG PS: 0 or 1, n (%)	63 (52.0)	11 (40.7)	5 (62.5)	47 (54.6)	0.31
**Diagnosis, n (%)**					
IgG kappa	54 (44.6)	14 (51.8)	5 (62.5)	35 (40.6)	0.36
IgG lambda	21 (17.3)	7 (25.9)	0	14 (39.5)	
IgA kappa	26 (21.4)	5 (18.5)	2 (25.0)	19 (22.0)	
IgA lambda	19 (15.7)	1 (3.7)	1 (12.5)	17 (19.7)	
Other	1 (0.8)	0	0	1 (1.1)	
**ISS, n (%)**					0.26
I	36 (29.7)	7 (25.9)	0	29 (33.7)	
II	29 (23.9)	8 (29.6)	2 (25.0)	19 (22.0)	
III	56 (46.2)	12 (44.4)	6 (75.0)	38 (44.1)	
**R-ISS, n (%)**					0.05
I	25 (20.6)	4 (14.8)	0	21 (24.4)	
II	65 (53.7)	16 (59.2)	6 (75.0)	43 (50.0)	
III	28 (23.1)	5 (18.5)	1 (12.5)	22 (25.5)	
Not available	3 (2.4)	2 (7.4)	1 (12.5)	0	
High risk cytogenetic abnormalities	23 (19.0)	2 (7.4)	2 (25.0)	19 (22.0)	0.1
β2-microglobulin (mg/L), median (range)	4.7 (1.2–50.3)	4.87 (1.6–18.1)	5.75 (3.2–13.5)	4.25 (1.2–50.3)	0.09
Albumin (g/dL), median (range)	3.3 (1.4–6.3)	3.2 (2.1–6.3)	3.1 (2.4–4.0)	3.3 (1.4–4.7)	0.81
LDH (U/L), median (range)	175 (60–1433)	185 (120–452)	161 (129–315)	171 (60–1433)	0.43
Creatinine (mg/dL), median (range)	0.89 (0.44–9.94)	0.89 (0.6–4.82)	0.91 (0.44–2.58)	0.83 (0.46–9.94)	0.43

IIMM: intact immunoglobulin multiple myeloma; ECOG: Eastern Cooperative Oncology Group; PS: performance status; ISS: international staging system; R-ISS: revised international staging system.

**Figure 1:**
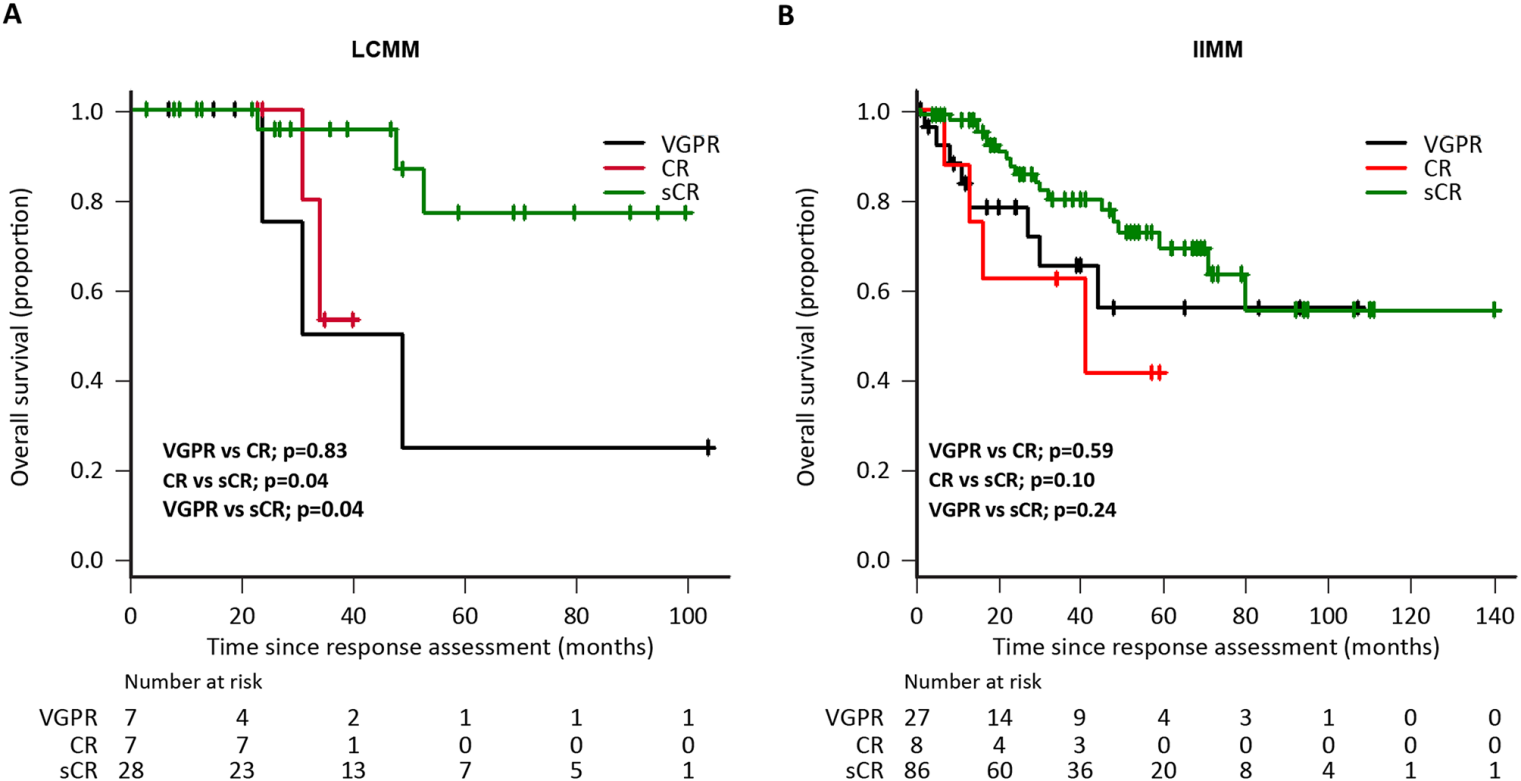
Overall survival according to best response, as defined by IMWG. (**A**) LCMM, (**B**) IIMM. IIMM, intact immunoglobulin multiple myeloma; IMWG, International Myeloma Working Group; LCMM, light chain multiple myeloma.

**Figure 2:**
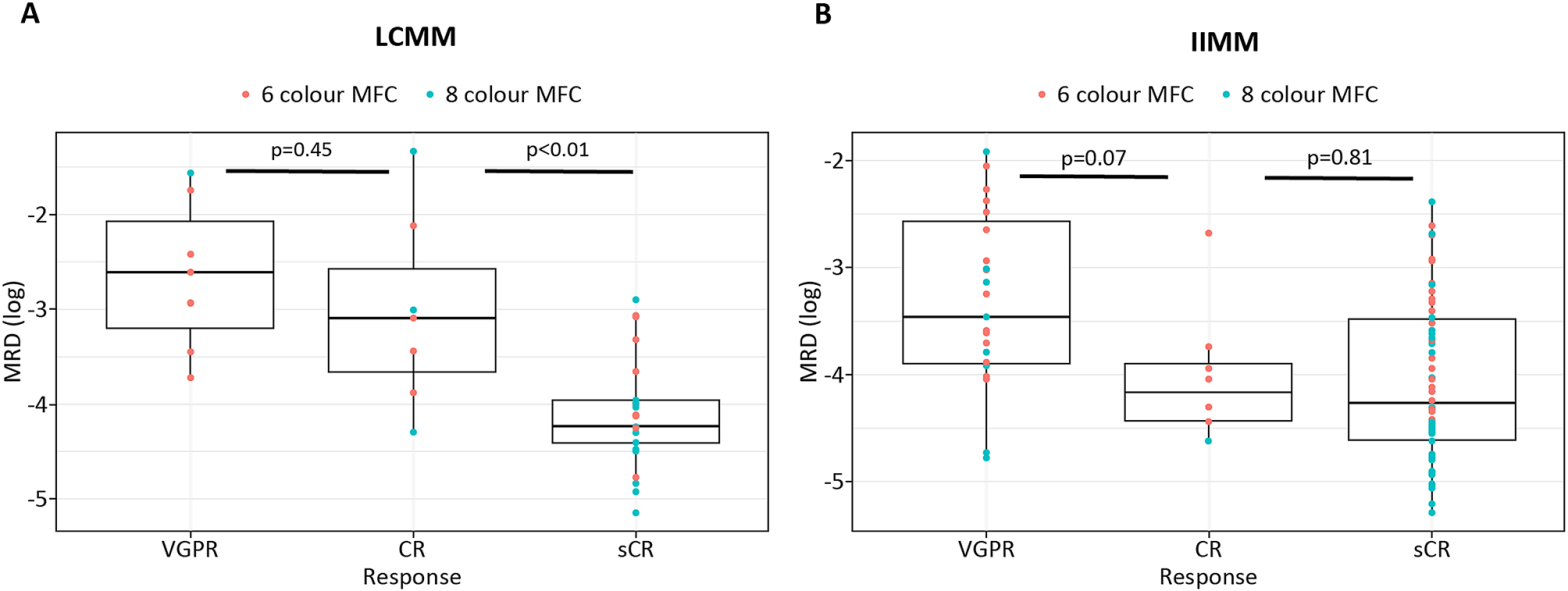
Measurable residual disease (MRD) according to best response as defined by IMWG. (**A**) LCMM, (**B**) IIMM. IIMM, intact immunoglobulin multiple myeloma; IMWG, International Myeloma Working Group; LCMM, light chain multiple myeloma.

The median level of paraprotein in patients with LCMM was 2375 mg (range 266–19,369). MRD assessment using 6-color flow cytometry was performed in 24 (57.1%) LCMM and 64 (52.8%) IIMM patients, whereas 8-color flow cytometry was performed in 18 (42.8%) LCMM and 57 (47.1%) IIMM patients. Fewer than 50 abnormal plasma cells were detected in 35 samples in IIMM (median abnormal plasma cells: 14, range: 1–44; median total analysed cells: 2,903,610, range: 511,854–9,859,391) and in 13 samples in LCMM (median abnormal plasma cells: 12, range: 1–49; median total analysed cells: 1,000,000, range: 105,161–7,167,456). Best responses of VGPR, CR, and sCR were achieved by seven (16.6%), seven (16.6%), and 28 (66.6%) patients with LCMM; and by 27 (22.3%), eight (6.6%), and 86 (71.0%) patients with IIMM, respectively. The median OS in LCMM patients with VGPR and CR was 58 and 41 months, respectively, while it was not reached among patients with sCR (VGPR vs. CR, *P* = 0.83; CR vs. sCR, *P* = 0.04; VGPR vs. sCR, *P* = 0.04; Fig. 1A). Contrastingly, the median OS was not reached in IIMM patients with VGPR and sCR, while it was 41 months among those achieving CR. These differences were not statistically significant between the response subgroups (VGPR vs. CR, *P* = 0.59; CR vs. sCR, *P* = 0.10; VGPR vs. sCR, *P* = 0.24; Fig. 1B). Thereafter, we compared MRD levels in each response category. In LCMM, sCR showed significantly deeper MRD than CR (median MRD in patients with CR vs. sCR: 7.9 × 10^−4^ vs. 5.6 × 10^−5^, *P* < 0.01); however, no difference in MRD was observed between patients with VGPR and CR (median MRD: 2.4 × 10^−3^ vs. 7.9 × 10^−4^, *P* = 0.45; Fig. 2A). In IIMM, MRD levels did not differ across patients with VGPR, CR, or sCR (VGPR vs. CR: 3.5 × 10^−4^ vs. 7.0 × 10^−5^, *P* = 0.07; CR vs. sCR: 7.0 × 10^−5^ vs. 5.4 × 10^−5^, *P* = 0.81; Fig. 2B). Among patients with LCMM or IIMM assessed for MRD using 8-color flow cytometry, the MRD negativity (< 1.0 × 10^−5^) rates were 5.5% (1/18 patients; all achieved sCR) and 12.2% (7/57 patients; all achieved sCR), respectively.

In patients with LCMM, the median OS was not statistically different among MRD-negative and MRD-positive patients because none of those who achieved ≥ VGPR died during the observation period. In IIMM, no MRD-negative and 10 MRD-positive patients died during the observation period; however, the median OS was not statistically different among MRD-negative and MRD-positive patients (median OS: not reached in both MRD-negative and MRD-positive patients, *P* = 0.20). Based on the ROC analysis, the MRD cut-off value used for OS analysis was 1.0 × 10^−4^ for both LCMM and IIMM (area under the curve [AUC] for LCMM: 0.71, 95% CI 0.54–0.88, sensitivity: 0.75, specificity: 0.61, Fig. S1A; AUC for IIMM: 0.64, 95% CI 0.53–0.75, sensitivity: 0.70, specificity: 0.55, Fig. S1B). The percentage of patients who achieved MRD < 1.0 × 10^−4^ was 50% (21/42) in LCMM (VGPR: 0/7, 0%; CR: 1/7, 14.2%; sCR: 20/28, 71.4%; *P* < 0.01) and 50.4% (61/121) in IIMM (VGPR: 6/27, 22.2%; CR: 5/8, 62.5%; sCR: 50/86, 58.1%; *P* < 0.01). Patients with LCMM who achieved MRD levels < 1.0 × 10^−4^ had a significantly longer OS than those who did not (median OS: not reached vs. 53 months, *P* = 0.02, Fig. S2A). In patients with IIMM who achieved MRD levels < 1.0 × 10^−4^ tended to have a longer OS than those who did not, but this difference was not statistically significant (median OS: not reached vs. 84 months, *P* = 0.16, Fig. S2B).

Multivariable Cox regression analysis for OS included the International Staging System (ISS), MRD < 1.0 × 10^−4^, and high-risk cytogenetic abnormalities as variables in addition to the response categories (VGPR, CR, and sCR) to elucidate whether achieving sCR showed a clearer improvement in OS than did other known risk factors. We included the ISS rather than the revised-ISS (R-ISS) because high-risk CA is included as a variable of R-ISS. Achieving sCR did not show risk reduction (hazard ratio [HR]: 0.92, 95% CI 0.33–2.60, *P* = 0.88) among patients with IIMM, but showed significant risk reduction (HR: 0.07, 95% CI 0.01–0.58, *P* = 0.01) among patients with LCMM (Table S3 and S4).

## Discussion

This study extends and updates our previous observations on the different depths of MRD among IMWG responses in patients with LCMM and IIMM. We previously showed that the attainment of sCR in LCMM indicated deeper MRD levels, but MRD levels in IIMM did not differ between CR and sCR^18^. The current study confirmed our previous observation that MRD levels during sCR were significantly lower than those during CR or VGPR in patients with LCMM. However, these levels were not different between CR and sCR in patients with IIMM. Additionally, in line with the MRD levels, we observed that in patients with LCMM, but not IIMM, achieving sCR versus CR prolonged survival. Quantitatively, residual MM plasma cell levels were 1-log higher in CR than in sCR in patients with LCMM (7.9 × 10^−4^ and 5.6 × 10^−5^, *P* < 0.01), whereas MRD levels during VGPR, CR, and sCR did not differ among patients with IIMM. The current definition of CR could explain these differences in patients with LCMM, which is a negative urine immunofixation indicating the disappearance of monoclonal light chain in the urine. However, the amount of light chain excreted in the urine is affected by the excretion and reabsorption capability of the kidneys, which in turn affects light chain metabolism^19^. As previously reported, serum FLC measurement was more accurate for monitoring and assessing tumour plasma cell burden in LCMM; the findings using this approach translated to better survival in LCMM patients with sCR and identification of the risk group in LCMM patients with CR. Nevertheless, serum FLC levels do not always reflect paraprotein levels in IIMM, offering an additional explanation for the lack of a difference in MRD levels between patients achieving VGPR, CR, or sCR. This may also explain why previous studies comparing survival between CR and sCR have conflicting results^5–10^.

A MRD cut-off level of 1.0 × 10^−4^ could discriminate OS in patients with LCMM, but not IIMM, in this study. Patients with IIMM with ≥ VGPR who had < 1.0 × 10^−4^ MRD tended to have a longer OS compared to those with higher MRD; this observation suggests that a deeper MRD level is needed for the prognostic relevance in IIMM. Nonetheless, a favourable predictive value of low sensitivity MRD negativity combined with conventional CR criteria are reported in a Medical Research Council Myeloma IX study^12^. Moreover, the survival benefit of achieving a negative MRD status at the MRD level of 1 × 10^−4^ has been confirmed in pooled clinical trials^20^. Furthermore, MRD negativity defined at > 10^−4^ to 10^−5^ surpasses the prognostic value of achieving PFS and OS, regardless of the type of treatment and patient risk group^21^. Similar to our study, this report also used an MRD value of 1.0 × 10^−4^ to discriminate survival. Recently, a highly prognostic predictive value was reported in patients achieving a deeper MRD response (< 1 × 10^−6^) irrespective of the presence or absence of poor prognostic features and high-risk CA using NGS^22^. In addition, an intense level of MRD negativity (< 1.0 × 10^−6^) is also reported as a strong prognostic indicator regardless of treatment, cytogenetic risk, and ISS stage^23^. Although NGS allows the detection of a deeper response that may be more relevant to longer PFS or OS, these methods are expensive and time consuming. Another limitation of detecting a deeper response using NGS is the requirement of a large number of bone marrow cells (at least 1.5 × 10^6^)^24^. Most patients with MM are of advanced age and are treated intensively; therefore, the resultant bone marrow hypoplasia makes the collection of enough cells for analysis challenging. However, sCR determined using the FLC assay could easily identify the subset of patients with LCMM with favourable prognosis in terms of MRD levels.

Limitations of this study include the relatively small sample size due to the inclusion of patients from a single institution, treatment heterogeneity, and its retrospective nature. Nevertheless, all patients in this study were treated using a combination of newer agents (e.g., bortezomib, lenalidomide, pomalidomide, carfilzomib, and daratumumab), and the background clinical characteristics were similar among patients with IIMM and LCMM who achieved response in each category. Another limitation is not using the EuroFlow, which is considered to be the standard for MRD analysis. Because the retrospective study period extended back to 2008, we instead used the less sensitive 6-color flow cytometry in a subset of patients diagnosed between 2008 and 2016. We also used DuraClone, which is a newly developed 8-color flow cytometry method that allows a sensitivity of 1.0 × 10^−5^. Although 8-color flow uses more antibodies than 6-color flow and the lower limit of detection used to define MRD negativity between 6- and 8-color flow cytometry differ, tumour-quantified MRD levels assessed by the two methods showed a good correlation. (r = 0.96, *P* < 0.01; Fig. S3). Yet another limitation stems from the fact that the most important aspect of MRD examination is an adequate marrow sampling and the number of bone marrow cells for analysis: this is not always feasible considering the advanced age and bone marrow hypoplasia of patients with MM post-treatment. Despite these limitations, our results suggest that achieving sCR using the IMWG definition should be interpreted with caution, especially in patients with IIMM whereas in patients with LCMM, sCR might be associated with a deeper response and favourable prognosis.

In conclusion, this study demonstrated variable MRD levels in patients with IIMM and LCMM within the same IMWG response categories. We also showed that achieving sCR represented a deeper response and favourable prognosis in patients with LCMM, but not in those with IIMM.

## Methods

### Study design and patients

The medical records of 206 patients with symptomatic MM (LCMM, n = 44; IIMM, n = 162) who were admitted at Kameda Medical Center, Japan, between April 2008 and December 2019 were reviewed. Treatment was selected at the physicians’ discretion and in agreement with the patient; nevertheless, all patients received doublet or triplet combinations of newer agents, such as bortezomib, thalidomide, or lenalidomide, as an initial treatment. Among them, 163 patients (79.1%) who achieved VGPR or better (≥ VGPR) were selected (LCMM, n = 42; IIMM, n = 121) for the analysis. Patients with monoclonal gammopathy of undetermined significance, smoldering MM, plasma cell leukaemia, and systemic amyloidosis were excluded. Patients without serum FLC or MRD data were also excluded. The best response during treatment according to the IMWG response criteria^4^ was adopted for assessment, and serum FLC and MRD evaluated at best response were used for analysis. In the determination of CR/sCR, we confirmed the disappearance of any soft tissue plasmacytomas by ^18^F-FDG PET/CT and the absence of clonal cells in bone marrow biopsy by immunohistochemistry in accordance with IMWG response guideline^4^. Because serum FLC did not necessarily correlate with the level of monoclonal immunoglobulin (Ig) levels in IIMM, the disappearance of the monoclonal band did not correlate with rFLC normalization. While FLC is a primary monoclonal protein in LCMM, its decrease is usually associated with rFLC normalization, although there are some exceptions. Therefore, we separated the patients into two groups, LCMM and IIMM, and compared the OS between each response category. To clarify whether achieving sCR contributed to longer survival, MRD levels between each response category were also evaluated to confirm whether sCR was a deeper response than CR in each group. We also analysed whether the reduction of MRD levels contributed to the longer survival in the included patients in real-world settings.

### Quantification of MRD

MRD assessment was performed using ethylenediaminetetraacetic acid-anticoagulated fresh bone marrow samples by staining with monoclonal antibodies using 6-color (2008–2016) or 8-color (2017–2019) flow cytometry, as previously described. In brief, 6-color flow cytometry analysis was performed using the CD38-PC7, CD138-APC, CD45-ECD, CD19-PE, CD56-PC5.5, and CD81-FITC antibody panels, based on the guidelines of the European Myeloma Network and its update^25,26^. The 8-color flow cytometry was performed using a DuraClone RE PC kit (Beckman Coulter, Brea, CA) by staining with prefixed dry reagents comprising CD138-APC, CD38-PB, CD56-APC-A750, CD19-PC5.5, CD45-KrO, CD200-PC7, CD81-FITC, and CD27-PE to identify abnormal plasma cells. Flow cytometric analysis was performed using the NAVIOS flow cytometer and Kaluza software v1.5a (Beckman Coulter). To ensure the clonality of the abnormal plasma cells, we stained the cytoplasmic Igκ FITC and Igλ PE in combination with CD38, CD45, CD138, and CD19 monoclonal antibodies, as supplementary. DuraClone is considered equivalent to EuroFlow in terms of MRD detection, with a lower limit of detection of 4.0 × 10^−6^^27,28^.

For quantification of MRD levels, we used the ‘Consensus Guidelines on Plasma cell Myeloma Minimal Residual Disease Analysis and Reporting’ with slight modifications. A minimum of 50 abnormal plasma cells were required for MRD analysis, and hemodilution was checked by the proportion of immature myeloid cells and erythroblasts in the bone marrow smear and clot histology of the samples. If < 50 abnormal plasma cells were detected, the numerator was set to 50 as a lower limit of quantification of abnormal plasma cells, and the total number of nucleated cells was used as a denominator; that is, for 20 abnormal plasma cells and 3 × 10^6^ total number of cells, the MRD would be 50/3 × 10^6^^29^. We also compared the correlation between 8-color flow cytometry and 6-color flow cytometry in the MRD quantification using the same samples simultaneously.

Serum FLC assay was performed using the Freelite assay (The Binding Site Group, Birmingham, UK), and normal serum rFLC (κ/λ) was defined as a range between 0.26 and 1.65^4^. MRD assessment and FLC analysis were simultaneously performed within a month after achieving the best response.

### Assessment of cytogenetic abnormalities

Cytogenetic abnormalities (CA) were analysed using interphase fluorescence in situ hybridisation (iFISH) including del(17p13), t(4;14)(p16;q32), t(14;16)(q32;q23), t(11;14)(q13;q32), del(13q), and gain of chromosome 1q. High-risk CA was defined as del(17p13), t(4;14)(p16;q32), and t(14;16)(q32;q23) according to the IMWG guidelines^30^. iFISH from 2016 to 2019 was performed with CD138-enriched bone marrow cells using CD138-coated magnetic MicroBeads (Miltenyi Biotech, San Diego, CA, USA)^30^ and from 2008 to 2015 with whole bone marrow cells. All iFISH analyses were performed before initiation of the treatment.

This study was approved by Kameda Medical Center institutional review board and performed in accordance with the Declaration of Helsinki. All patients provided written informed consent.

### Statistical analyses

Comparison of continuous variables between three or more sets was performed using the Kruskal–Wallis test. Comparisons of categorical variables were performed using Fisher’s exact test. Survival analysis was performed using the Kaplan–Meier method. Survival of groups was compared using the log-rank test. *P* values between three or more sets were adjusted using the Benjamini and Hochberg method for controlling the false discovery rate^31^. OS was calculated from the time point of achieving the best response to that of death from any cause (landmark analysis) to mitigate the potential confounding factors by guarantee-time bias on the basis of best response. Furthermore, receiver operating characteristic (ROC) curve analysis was performed to determine the optimal MRD level for prolonged OS in LCMM and IIMM patients. The Cox regression hazard model was used to assess the association between variables of known risks and OS. All *P* values were two-sided, and *P* < 0.05 was considered statistically significant. All statistical analyses were performed using R (version 4.0.2; R Foundation, Vienna, Austria).

## Supplementary Information

Supplementary Information 1.

Supplementary Information 2.

Supplementary Information 3.

Supplementary Information 4.

Supplementary Information 5.

Supplementary Information 6.

Supplementary Information 7.

## Data Availability

The datasets generated during and analysed during the current study are available from the corresponding author on reasonable request.
