# Supplementary material for: Quantification of measurable residual disease in patients with multiple myeloma based on the IMWG response criteria
Source: Sci Rep. 2021 Jul 22;11:14956. doi: 10.1038/s41598-021-94191-8 (PMC8298479; doi:10.1038/s41598-021-94191-8)
Supplement: Supplementary file 1 — Supplementary Information 1. [file 41598_2021_94191_MOESM1_ESM.docx]

**SUPPLEMENTARY FIGURE LEGENDS:**

**Fig S1:** Receiver operating characteristic curve analysis to define the cut-off value of MRD. (A) LCMM, (B) IIMM. IIMM: Intact immunoglobulin multiple myeloma, LCMM: Light chain multiple myeloma, MRD: Measurable residual disease.

**Fig S2:** Overall survival according to MRD≥ 10^4^ and MRD< 10^4^. (A) LCMM, (B) IIMM. IIMM: Intact immunoglobulin multiple myeloma, LCMM: Light chain multiple myeloma, MRD: Measurable residual disease

**Fig S3:** Scatter plot of MRD analysed by 6-or 8-color FCM showing good concordance. MRD; Measurable residual disease

**SUPPLEMENTARY TABLE CAPTIONS:**

**Table SI:** First-line treatment regimen and ASCT percentage in patients with LCMM (A). First-line treatment regimen and ASCT percentage in patients with IIMM (B), ASCT: Autologous stem cell transplantation, IIMM: Intact immunoglobulin multiple myeloma, LCMM: Light chain multiple myeloma

**Table SII:** Multivariate analysis of factors affecting OS (A) LCMM, (B) IIMM, IIMM: Intact immunoglobulin multiple myeloma, LCMM: Light chain multiple myeloma
