# Supplementary figures and images for: Quantification of measurable residual disease in patients with multiple myeloma based on the IMWG response criteria
Source: Sci Rep. 2021 Jul 22;11:14956. doi: 10.1038/s41598-021-94191-8 (PMC8298479; doi:10.1038/s41598-021-94191-8)

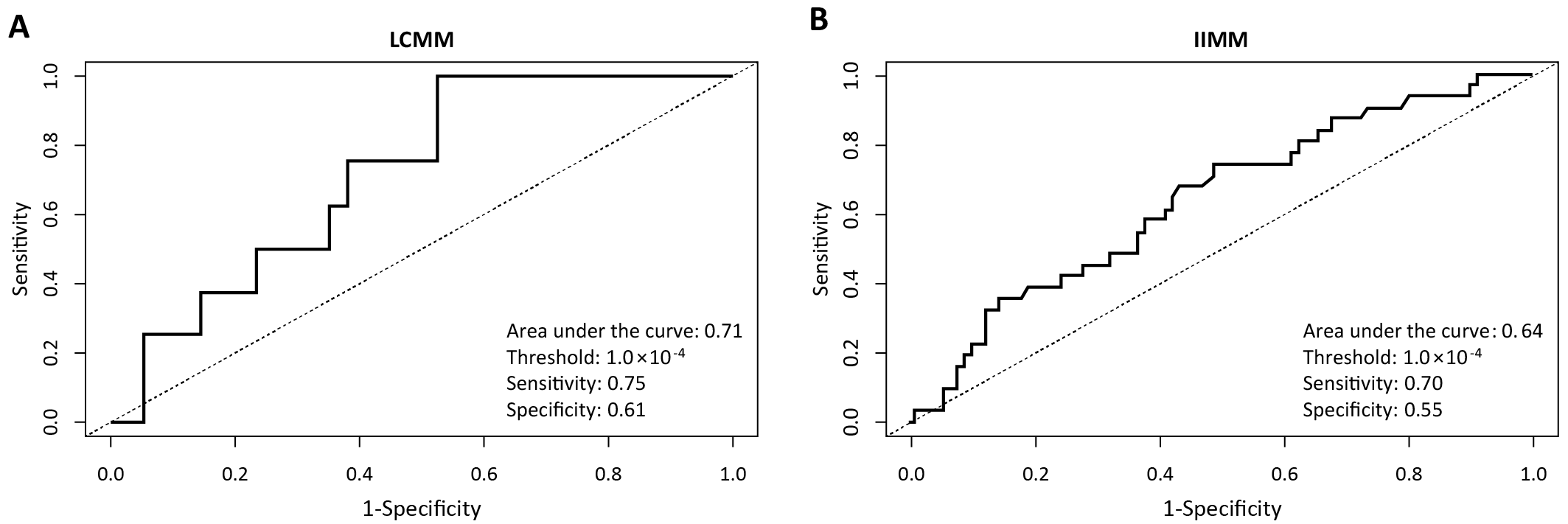

Supplement: Supplementary file 2 — Supplementary Information 2. [file 41598_2021_94191_MOESM2_ESM.tif]

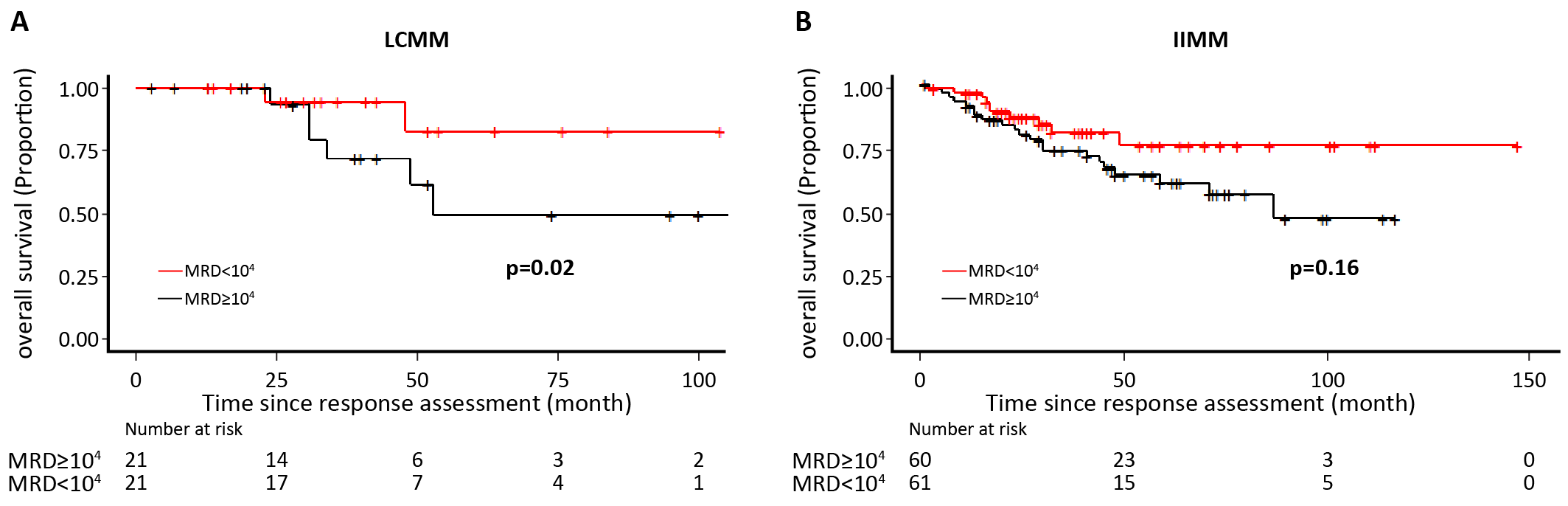

Supplement: Supplementary file 3 — Supplementary Information 3. [file 41598_2021_94191_MOESM3_ESM.tif]

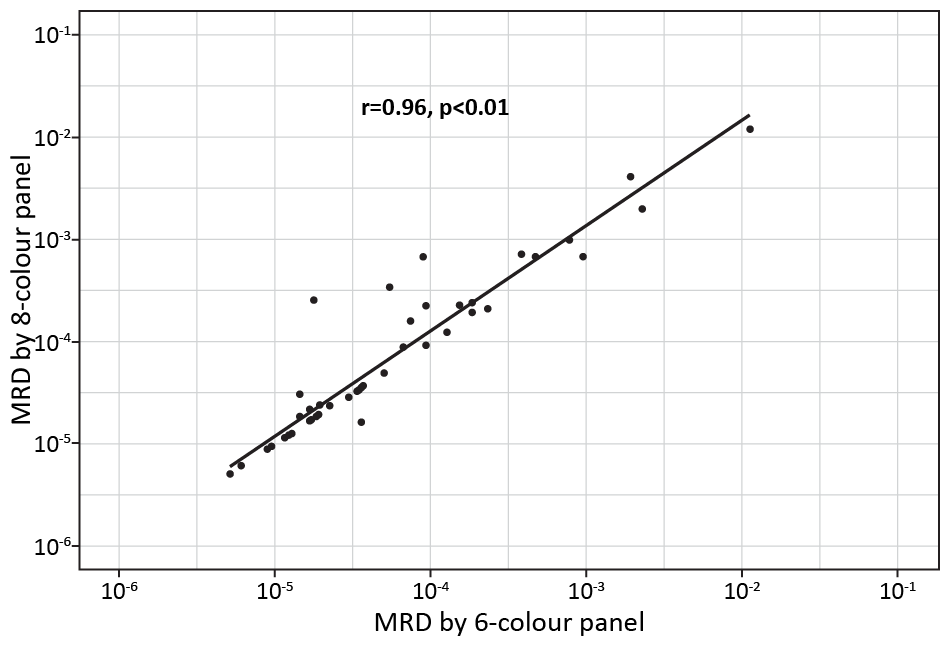

Supplement: Supplementary file 4 — Supplementary Information 4. [file 41598_2021_94191_MOESM4_ESM.tif]

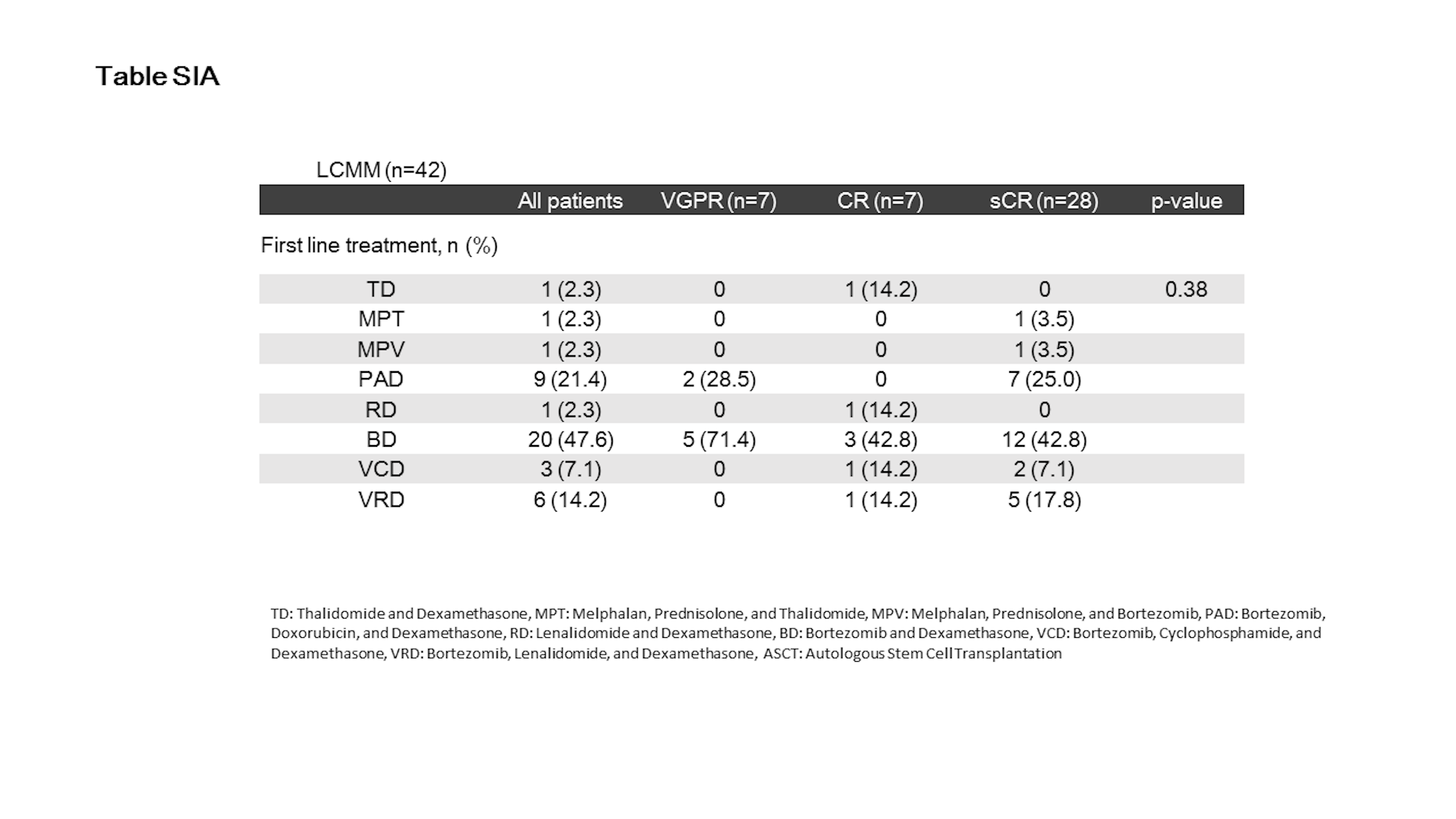

Supplement: Supplementary file 5 — Supplementary Information 5. [file 41598_2021_94191_MOESM5_ESM.tif]

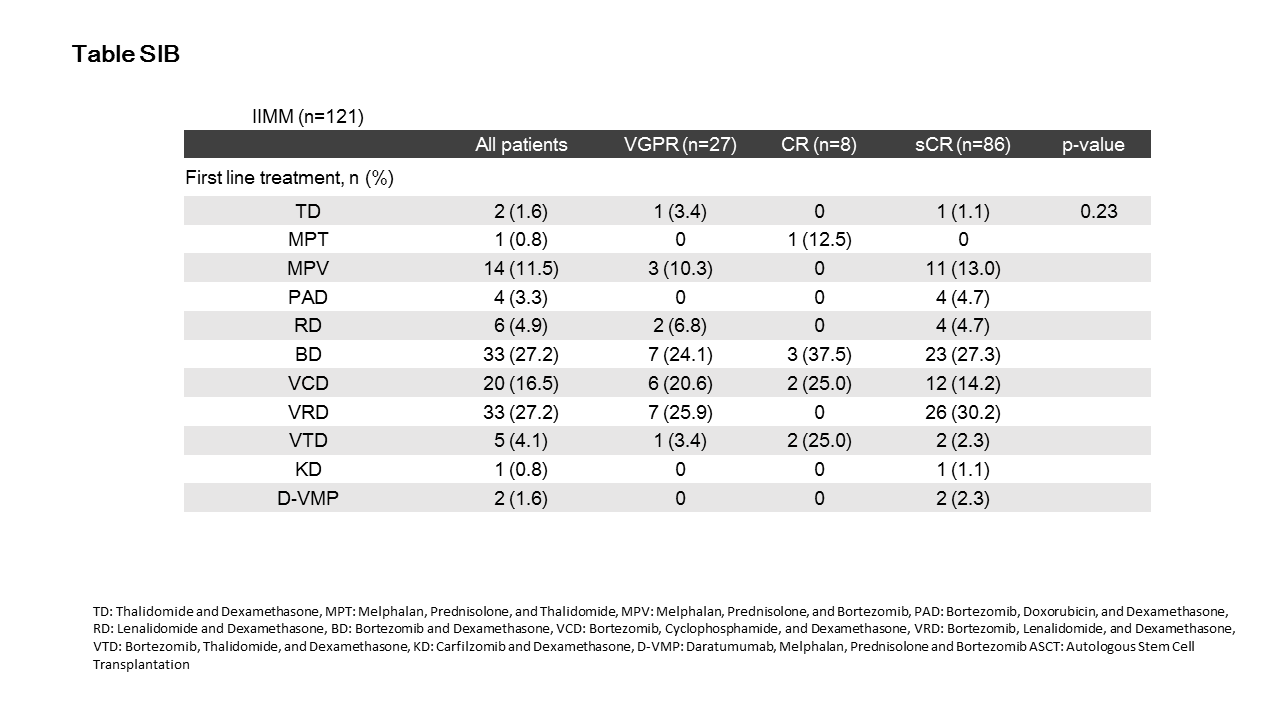

Supplement: Supplementary file 6 — Supplementary Information 6. [file 41598_2021_94191_MOESM6_ESM.tif]

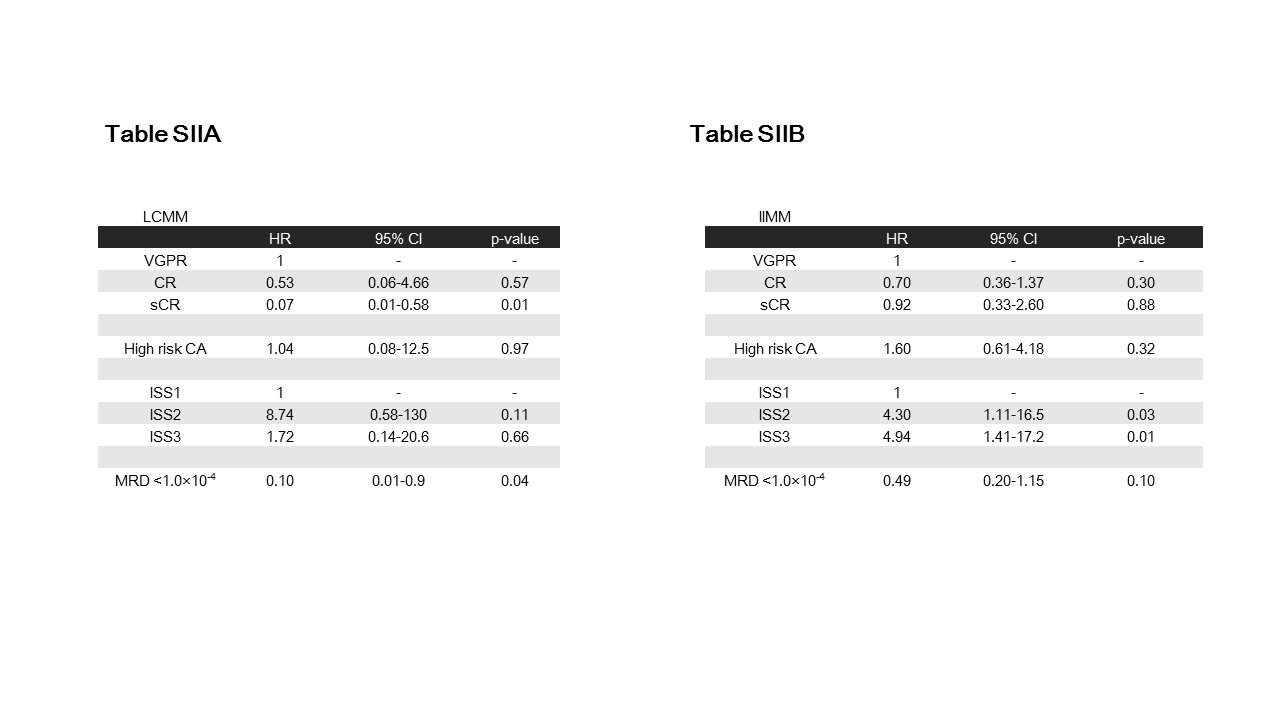

Supplement: Supplementary file 7 — Supplementary Information 7. [file 41598_2021_94191_MOESM7_ESM.tif]
